# Short-Term Evaluation of Bone–ACL–Bone Complex Allograft in ACL Reconstruction in a Rabbit Model

**DOI:** 10.3390/jcm12227057

**Published:** 2023-11-13

**Authors:** Yulei Liu, Janice Havasy, Samuel Green, Xiang-Hua Deng, Daoyun Chen, Alexander Piacentini, Scott A. Rodeo

**Affiliations:** 1 Department of Sports Medicine, Peking University Third Hospital, Beijing 100191, China; 2 Orthopedic Soft Tissue Research Program, Hospital for Special Surgery, New York, NY 10021, USA

**Keywords:** allograft, anterior cruciate ligament reconstruction, bone–ACL–bone, animal model, healing

## Abstract

The study is to evaluate incorporation of a bone-anterior cruciate ligament-bone (B-ACL-B) allograft in anterior cruciate ligament (ACL) reconstruction in a rabbit model. A total of 61 New Zealand white rabbits were used, with 23 donor rabbits for harvesting B-ACL-B allografts and 38 recipient rabbits undergoing unilateral ACL reconstruction with B-ACL-B allograft. Animals were euthanized for biomechanical testing, micro-computed tomography examination, histological analysis, multi-photon microscopy and transmission electron microscopy testing at 2, 4 and 8 weeks after surgery. Gross inspection and radiographs confirmed the intact ACL allograft in the proper anatomic position. Progressive healing occurred between the bone block and the bone tunnel as demonstrated by a gradual increase in average bone volume fraction and total mineral density at 4 and 8 weeks. Histological analysis showed new bone formation at the bone block–tunnel interface, with maintenance of the native ACL enthesis. Ultrastructural analysis demonstrated the maintenance of overall collagen matrix alignment, while there was repopulation with smaller diameter collagen fibrils. There was no significant difference between 4 and 8 weeks in mean failure force (*p* = 0.39) or stiffness (*p* = 0.15) for the B-ACL-B allografts. This study demonstrates the restoration of the normal anatomy of the ACL and progressive graft incorporation and remodeling using a B-ACL-B allograft for ACL reconstruction in the rabbit knee.

## 1. Introduction

Anatomic anterior cruciate ligament (ACL) reconstruction is defined as the restoration of the native insertion sites, tissue dimensions, and collagen orientation. However, the microstructure and composition of standard tendon grafts used for ACL reconstruction do not replicate the highly specialized microscopic and ultrastructural anatomy of the ACL. The ACL contains fibers of varying lengths that undergo reciprocal tightening and loosening with knee motion, allowing for the maintenance of anterior tibial stability, and the complex interaction between collagen fibril microarchitecture and the associated non-collagenous matrix imparts the unique material properties to this ligament [1,2]. This complex microstructure and composition of the native ACL is not re-established following ACL reconstruction, as the microscopic anatomy and composition of tendon grafts used for ACL reconstruction is very different than that of the native ACL. Furthermore, the biologic process of graft incorporation involves revascularization, cellular repopulation and matrix remodeling, which results in a tissue with a structure and composition that differs from the native ACL [3,4,5]. 

Another critical limitation in current ACL reconstruction techniques is the graft attachment site to bone. The native ACL insertion site microstructure, with intervening zones of mineralized and unmineralized fibrocartilage, does not reform during healing of a tendon graft in a bone tunnel. Rather, the tendon graft heals to the bone tunnel with an intervening zone of fibrovascular scar tissue, which has inferior material properties compared to the native insertion site [4,5,6,7]. The lack of reformation of the normal gradient in collagen fiber ultrastructure, mineralization, and matrix protein composition across the insertion site renders the graft susceptible to micromotion at the bone–tendon interface, leading to the potential for recurrent knee laxity. 

These limitations of contemporary ACL reconstruction techniques using a standard tendon graft in a bone tunnel suggest the potential to improve outcomes using a bone–ACL–bone (B-ACL-B) allograft for ACL reconstruction. This approach would allow for the replacement of the ACL with a graft with the appropriate collagen fibril macrostructure and microstructure to reproduce the normal length pattern and mechanical properties of the native ligament structure. Furthermore, this approach would allow for the implantation of a graft with a native ligament-to-bone insertion site, where fixation would rely on predictable bone-to-bone healing with the preservation of the native ligament insertion site anatomy. An obvious challenge using a B-ACL-B allograft is the need to match the graft length (femur-to-tibia) to the recipient knee. 

ACL reconstruction using a B-ACL-B graft has not been used clinically, but several prior pre-clinical studies (goat and dog models) have examined the healing process of a B-ACL-B allograft after ACL reconstruction. These studies have generally focused on the healing of the bone block in a bone tunnel, with some studies demonstrating complete bone union, while other studies reported delayed and incomplete bone plug healing [8,9,10,11,12,13,14,15]. For example, Vasseur evaluated frozen bone–ACL–bone allografts in dogs and reported relatively poor healing at the bone plug interface, which they felt was due to the presence of antibodies against donor dog leukocyte antigen in the synovial fluid [15]. These prior studies have largely focused on bone plug incorporation, with minimal information about the collagen fiber architecture of the graft mid-substance. A rabbit biomechanical study showed maximum failure forces of 23% of the native ligament at 16 weeks postoperatively [13]. Taken together, there is a paucity of experimental data on B-ACL-B allograft reconstruction in the recent literature, and many of these studies were completed over 25 years ago, suggesting the need for further evaluation of B-ACL-B graft incorporation. 

We hypothesize that a B-ACL-B allograft may be the ideal material for ACL reconstruction due to biomechanical and anatomic considerations. In addition to matching graft geometry, the concept of replacing the ACL with a B-ACL-B graft relies on two fundamental principles: 1. Maintenance of the collagen fiber architecture of the implanted graft during the graft remodeling process, and 2. Secure healing of the bone block in a bone tunnel. Thus, the purpose of the present study was to evaluate the early healing of the transplanted B-ACL-B allograft in a rabbit model, using biomechanical, radiographic, micro-computed tomography (μCT) and histological analyses.

## 2. Methods

### 2.1. Study Design

**Figure 1:**
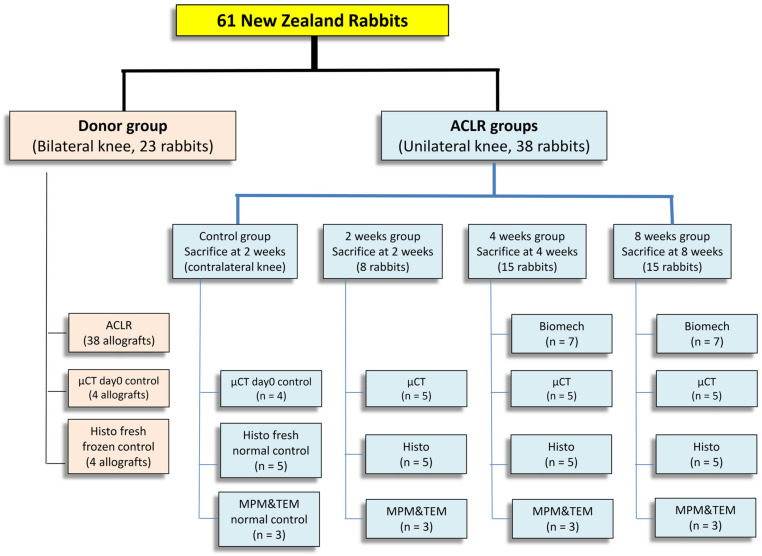
Study design and surgical groups. ACLR: Anterior cruciate ligament reconstruction. μCT: micro-computed tomography, Histo: histology, Biomech: biomechanics, MPM: multi-photon microscopy, TEM: transmission electron microscopy.

This study was approved by the Institutional Animal Care and Use Committee. A total of 61 skeletally mature New Zealand white rabbits were used, with 23 donor rabbits used for the harvesting of bilateral B-ACL-B donor allografts, while 38 recipient rabbits underwent unilateral ACL resection followed by reconstruction with B-ACL-B allografts. The allografts were harvested from male rabbits, while the recipient rabbits were female. Rabbits were randomly euthanized at 2, 4 and 8 weeks, and the tissue was harvested for histology (*n* = 5), μCT scanning (*n* = 5), biomechanical testing (*n* = 7), multi-photon microscopy (MPM) and transmission electron microscopy (TEM) (*n* = 3) (Figure 1). Contralateral normal knees were used for time zero μCT analysis (*n* = 4); time zero B-ACL-B graft histology (*n* = 4); and histology, MPM, and TEM of the native ACL (*n* = 5). 

### 2.2. Surgical Procedure

#### 2.2.1. Femur–ACL–Tibia Allograft Harvest and Preparation

The donor animals had similar body weight to the recipient rabbits. Donor femur–ACL–tibia allografts were harvested from male rabbits immediately after euthanasia. The bone blocks on the femoral and tibial side were trimmed to a length of 7 and 5 mm, respectively, and a diameter of 3.5 mm. After rinsing with a 5% solution of penicillin three times, the grafts were stored at −80 °C for 3–5 weeks prior to transplantation. 

#### 2.2.2. ACL Reconstruction Procedure

**Figure 2:**
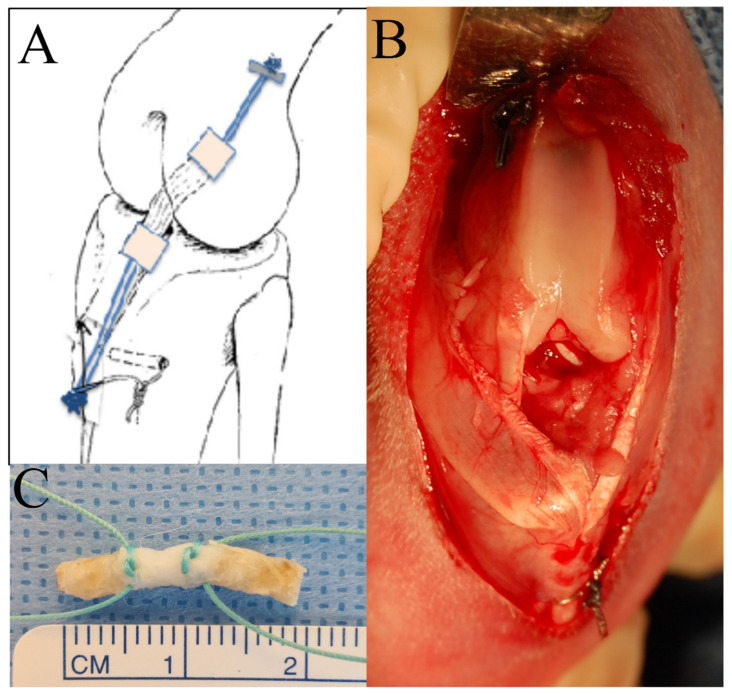
(**A**) Illustration of reconstruction plan for the bone-anterior cruciate ligament-bone (B-ACL-B) allograft. (**B**) Gross appearance after allograft implantation. (**C**) Typical appearance of harvested B-ACL-B allograft with passing sutures.

The graft was thawed prior to surgery and a 2.0 Ethibond suture (Ethicon, Somerville, Bridgewater, NJ, USA) was placed at the bone–ACL interface (Figure 2C). After the native ACL was resected, the femoral tunnel was drilled with a 1.1 mm K wire from the ACL origin, followed by a 3.5 mm cannulated drill for a 7 mm deep socket. The tibial tunnel was drilled over a 1.1 mm K wire using a 3.5 mm drill from the outside in, entering the joint at the footprint of the native ACL. Suspensory fixation was used on the femoral side using a 6 mm clip, and then the sutures on tibial side were fixed to the tibia using a transosseous 3-0 steel wire passed through the anterior crest of the tibia with the knee at 50 degrees of flexion with manual tension applied. (Figure 2). The rabbits were allowed ad libitum activity in individual cages and received routine buprenorphine analgesia for 72 h postoperatively.

#### 2.2.3. Radiographic Imaging

Anterior–posterior and lateral images of whole-knee specimens were acquired for confirmation of the position of the allograft bone block and fixation devices for each rabbit using high-resolution radiography (Faxitron Bioptics, LLC, Tucson, AZ, USA).

#### 2.2.4. Biomechanical Testing

**Figure 3:**
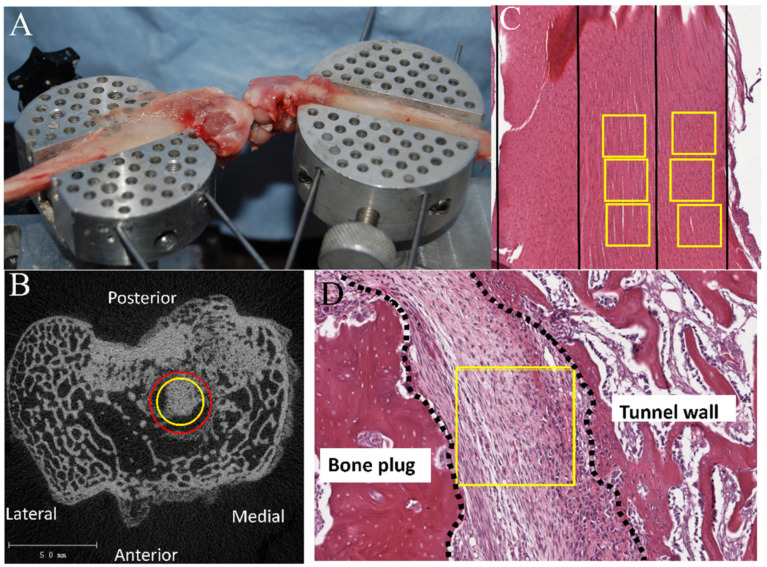
(**A**) Biomechanical setup. (**B**) Measurement of new bone formation between bone block and tunnel wall. The region between the yellow circle (diameter: 3 mm, same as bone block diameter) and the red circle (diameter: 3.5 mm, same as tunnel diameter) represents the interface between the bone block and the tunnel wall. (**C**) Measurement of cellularity in the central area and periphery of ligament. Magnification: 4×. Yellow boxes represent 3 consecutive regions of interest of 300 × 300 μm established centrally and peripherally along the mid-portion of the ACL respectively. (**D**) Measurement of cellularity in the interface between bone block and tunnel wall. Magnification: 10×. Yellow box represents a region of interest of 300 × 300 μm set at the bone block–tunnel interface.

All specimens underwent a single freeze–thaw cycle at −80 °C until the time of testing. The graft fixation sutures were removed. The femur–ACL graft–tibia complexes were fixed in a custom-designed Materials Testing system (Figure 3). After applying 3 cycles of 1N cyclic preload, a tensile failure test was carried out at an elongation rate of 10 mm/min. 

#### 2.2.5. μCT Analysis 

The Scanco μCT 35 (Scanco Medical, Bassersdorf, Switzerland) system and software (DECwindows Motif 1.6; Hewlett-Packard, Palo Alto, CA, USA) were used for μCT scan, viewing, image analysis, and 3-dimensional reconstruction, by identifying a cylindrical volume of interest with a diameter of between 3 mm (bone block diameter) and 3.5 mm (bone tunnel diameter) (Figure 3). Newly formed bone between the bone block and the tunnel was measured as bone volume fraction (BVF, bone volume/total volume (voxels/mm^3^)).

#### 2.2.6. Histology and Histomorphometry

After formalin fixation, decalcification, and paraffin embedding, tissues were sectioned along the bone tunnel in the sagittal plane into 5 μm thick slices and stained with hematoxylin and eosin (H&E) and Safranin-O. Images were obtained using light and polarized light microscopy (Eclipse E800; Nikon, Melville, NY, USA) and a SPOT RT camera (Diagnostic Instruments, Sterling Heights, MI, USA). 

For the semi-quantitative analysis of cellularity, cell number was counted within a region of interest (ROI) of 300 × 300 μm set at the bone block–tunnel interface using QuPath software (Kingston, ON, Canada) [16]. The cell density (/10,000 µm^2^) was calculated by dividing the total cell number by the area, and the average in 5 separate ROIs were recorded. For ligament mid-substance analysis, 3 consecutive ROIs of 300 × 300 μm were established centrally and peripherally along the mid-portion of the ACL (Figure 3), and the averages were recorded separately. All histomorphometric analyses were performed in a blinded fashion.

### 2.3. Multi-Photon Microscopy and Transmission Electron Microscopy

MPM images were used to quantify collagen fibril organization by measuring the ratio between the peak intensities at the primary angles of alignment among the fibrils and the intensity at an orthogonal angle. Electron microscopy was used to measure collagen fibril diameters. Details of the methodology are presented below. 

Specimens were fixed overnight in 4% paraformaldehyde (PFA). Bone blocks on the samples were glued to glass slides to retain the in vivo shape and orientation of the ACL. Images were taken using an upright Leica SP8 with DIVE optics and a SpectraPhysics Mai Tai DeepSee laser (Spectra Physics, Milpitas, CA, USA). The excitation wavelength was 780 nm with second harmonic generation (SHG) detection between 380 nm and 412 nm. All images were 512 × 512 pixels, taken at 5×/0.15 and 10×/0.4 dry objectives. Fast Fourier Transform (FFT) analysis on the 10× images was used following the Taylor et al. protocol in Fiji Image J [17]. The % ratio from the orthogonal is a ratio between the peak intensities at the primary angles of alignment among the fibrils to the intensity at an orthogonal angle. Each image was analyzed 3 times in order to capture an average and representative collagen organization. 

After MPM, the same samples were prepared for TEM by further fixation using PFA and glutaraldehyde for a week prior to embedding. Samples were cut to 500 nm thickness and imaged on a JEM-1400 electron microscope at 50,000× magnification with an accelerating voltage of 100 kV. Using ImageJ, the perimeter was traced around each individual fibril for measurement of collagen fibril diameter (average 442 fibrils measured per slide).

### 2.4. Statistical Analysis 

All data are presented as means ± standard deviation. One-way analysis of variance (ANOVA) with the post hoc Tukey or Dunnett’s multiple comparisons test was used to analyze differences among groups. For all analyses, *p* < 0.05 was considered significant. All statistical analyses were performed using SPSS version 21.0 (IBM, Armonk, NY, USA).

## 3. Results

### 3.1. Macroscopic Observation and Radiographic Imaging

**Figure 4:**
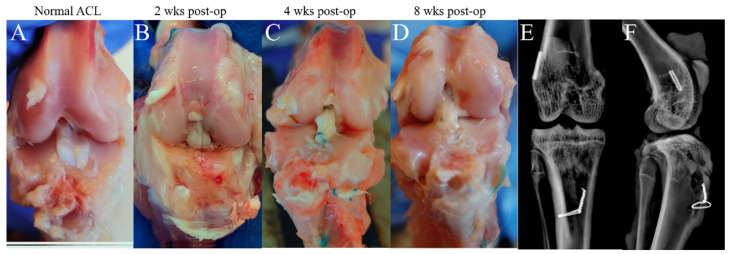
(**A**–**D**) Gross inspection of the specimen at all time points demonstrated intact anterior cruciate ligament allograft without rupture. (**E**,**F**) Radiographic imaging showing satisfactory fixation without bone block pull-out or fracture.

A gross inspection of the specimens at all time points showed intact grafts with normal anatomical appearance and well-fixed bone blocks in all knees. (Figure 4A–D) There was no instance of graft rupture, bone block fracture, or bone block pull-out from the tunnel. Radiography confirmed that all femoral and tibial tunnels were in a satisfactory position and that all clips and wires remained in a stable position (Figure 4E,F).

### 3.2. Biomechanical Analysis

**Figure 5:**
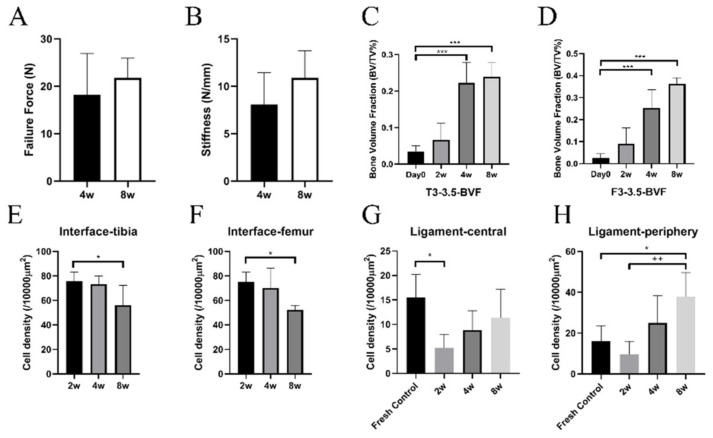
(**A**) Failure force. (**B**) Stiffness. (**C**) Bone volume fraction (BVF) in the femoral tunnels. (**D**) BVF in the tibial tunnels. (**E**) Cell density results at the tibial tunnel–bone block interface. (**F**) Cell density results at femoral tunnel–bone block interface. (**G**) Cell density of ACL central substance. (**H**) Cell density of ACL periphery. (*: *p* < 0.05, “++”: *p* < 0.01, ***: *p* < 0.001).

There was no significant difference in mean failure force between 4 weeks and 8 weeks (*p* = 0.39, Figure 5). There was also no significant difference in mean stiffness between 4 weeks and 8 weeks (*p* = 0.15). Twelve (85.7%) grafts failed by avulsion fracture near the ligament–bone insertion site on the femoral side, one (7.1%) on the tibial side, and one graft (7.1%) failed at the ligament mid-substance.

### 3.3. μCT Analysis

Femoral Tunnels: μCT quantification of newly formed bone in the femoral tunnels demonstrated progressive healing, with a significant increase in BVF at both 4 and 8 weeks at the bone block–bone tunnel interface compared to time zero (both *p* < 0.001) (Figure 5). There was a significant increase in total mineral density (TMD) at 2, 4, and 8 weeks compared to time zero (*p* = 0.03, 0.024 and 0.005, respectively).

Tibial Tunnels: There was progressive healing between the bone block and the bone tunnel, with a significant increase in BVF at 4 and 8 weeks compared to time zero (*p* = 0.004 and *p* < 0.001, respectively), but there was no significant difference in TMD postoperatively with time (*p* = 0.333) (Figure 5). 

### 3.4. Histological Analysis

#### 3.4.1. Bone Plug-to-Tunnel Healing

**Figure 6:**
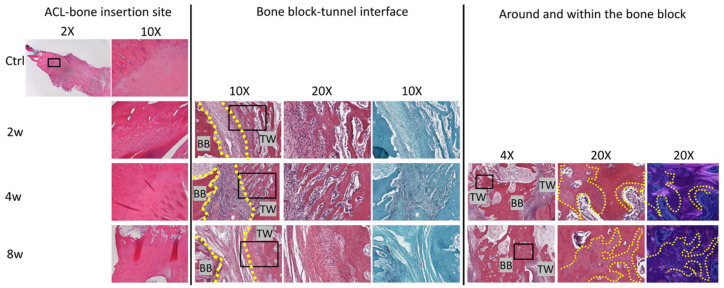
Native anterior cruciate ligament (ACL) –bone insertion site. New bone formation at the bone block–tunnel interface, and around and within the bone block. Hematoxylin and eosin staining of ACL–bone insertion site demonstrates maintenance of the histologic microstructure and composition of the native ACL insertion to bone at 2, 4 and 8 weeks. Yellow dot lines show the bone block–tunnel interface and the region of new bone. The regions in black boxes are shown in the following photos with magnification of 20×. BB: bone block, TW: tunnel wall.

There was new trabecular bone formation between the allograft bone block and the recipient bone tunnel in both femoral and tibial tunnels at all time points. Abundant osteocytes and osteoblasts were present in this newly formed woven bone and in the advancing ossification front. At the bone block–tunnel interface, there was a region of connective tissue containing fibroblast-like cells, vessels, some adipose tissue, osteoclasts, scattered inflammatory cells, and less differentiated cells (Figure 6). Cellularity in the interface tissue decreased over time, with significantly lower cellularity in both the femoral and tibial tunnel interfaces at 8 weeks compared to 2 weeks (2 weeks: *p* = 0.014 for both tunnels), indicating the resolution of the inflammatory stage of healing (Figure 5). There was no fibrocartilage formation or Safranin-O staining showing the accumulation of proteoglycans at the tunnel interface, suggesting that healing between the bone block and bone tunnel was achieved mainly by direct (intramembranous) ossification. 

#### 3.4.2. Bone Plug Remodeling

**Figure 7:**
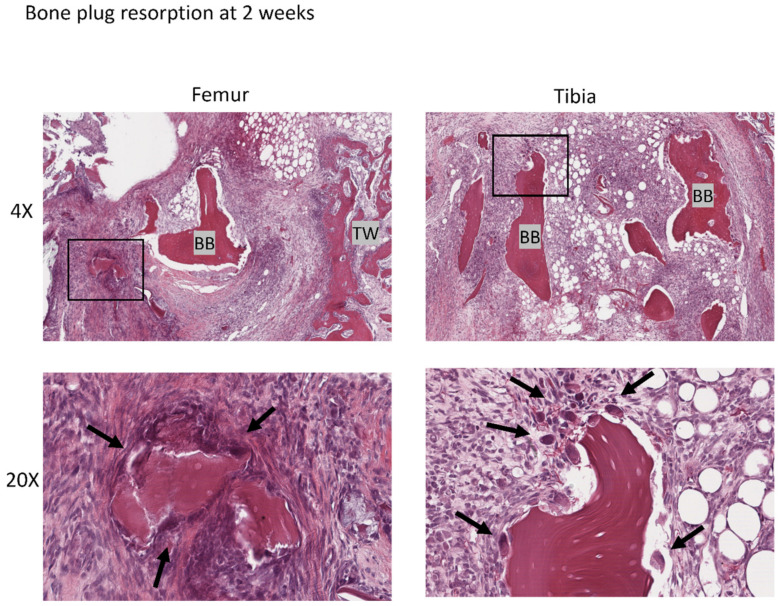
Bone block remodeling at 2 weeks. BB: bone block, TW: tunnel wall. Arrow: osteoclasts. There was a densely cellular connective tissue around the bone fragments, with osteoclast-mediated scalloping on the perimeter and proliferation showing areas of bone resorption. The regions in black boxes are shown in the photos below with magnification of 20×.

At the 2 week time point, the implanted bone block showed evidence of resorption, with osteoclast-mediated scalloping on the perimeter, along with areas of immature new woven bone formation (Figure 7). At 4 weeks, bony bridges can be seen connecting the host bone with the implanted bone block. At 8 weeks there was further integration of the implanted bone block into both femoral and tibial bone tunnels. Polarized microscopy demonstrated collagen fibril continuity between the bone block and the newly forming bone along the tunnel (Figure 6). There was generally maintenance of the histologic microstructure and composition of the native ACL insertion to bone, with zones of unmineralized and mineralized fibrocartilage separated by a tidemark (Figure 6). 

For the bone block–tunnel interface, the yellow dotted lines show the edge of bone block and tunnel wall. H&E staining shows new trabecular bone forming along the tunnel walls, with abundant osteocytes and osteoblasts present among the newly formed woven bone and in the advancing ossification front. At the bone block–tunnel interface, there was a region of connective tissue containing fibroblast-like cells, vessels, some adipose tissue, osteoclasts, and scattered inflammatory cells. Safranin O staining (right panel) did not show fibrocartilage formation or the accumulation of proteoglycans at the tunnel interface. 

For the new bone formation around and within the bone block, the yellow dotted lines outline the interface of newly formed bone. Bony bridges can be seen connecting the host epiphyseal bone with the implanted bone at 4 weeks. At 8 weeks, there is new bone formation within the bone block. Polarized microscopy demonstrates collagen fibril continuity between the bone block and the newly forming bone along the tunnel. 

#### 3.4.3. ACL Mid-Substance Remodeling 

**Figure 8:**
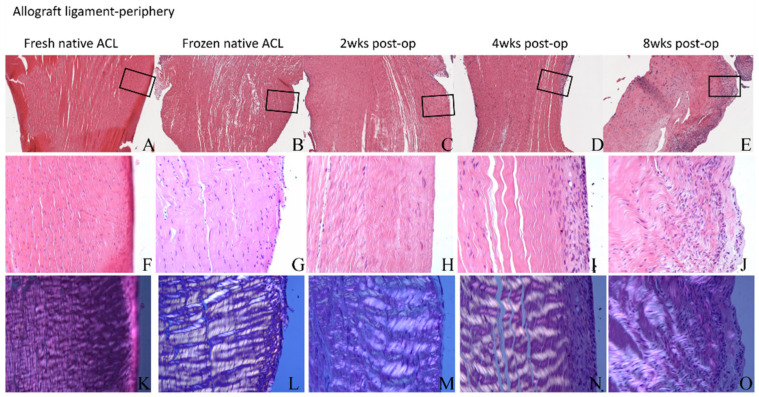
Anterior cruciate ligament (ACL) mid-substance histology. (**A**–**E**) Hematoxylin and eosin (H&E) staining, 4×. (**F**–**J**) H&E staining, 20×. H&E staining shows cellular infiltration into the periphery of the ACL, resulting in increased cell density at 8 weeks compared to fresh and frozen control ACL and 2 weeks group. (**K**–**O**) Polarized microscopy shows decreased birefringence at 8 weeks in the peripheral aspect of the graft in the areas of cellular infiltration, consistent with loss of collagen fibril organization due to matrix remodeling. The regions in black boxes are shown in the photos below with magnification of 20×.

The central ligament substance showed diminished cellularity at each time point compared to a native fresh ACL (Figure 8). The cell density at 2 weeks was significantly decreased compared to the native ACL (5.23 ± 2.76/10,000 mm^2^ vs. 15.5 ± 4.68/10,000 mm^2^, *p* = 0.011). Overall collagen matrix organization was maintained, with well-oriented collagen fibers and no signs of collagen matrix fragmentation or degradation. There was obvious cellular infiltration into the graft, beginning along the graft periphery. The cell density in both the central and peripheral areas of the graft increased with time, with peripheral cellularity at 8 weeks being significantly higher compared to the native ACL (*p* = 0.013) and the 2 week groups (*p* = 0.001), indicating that the soft tissue remodeling process starts from the ligament periphery (Figure 5). Polarized microscopy showed decreased birefringence at 8 weeks in the peripheral aspect of the graft in the areas of cellular infiltration, which is consistent with loss of collagen fibril organization due to matrix remodeling and new matrix synthesis (Figure 8).

### 3.5. Ultrastructural Evaluation with Multi-Photon Microscopy and TEM

**Figure 9:**
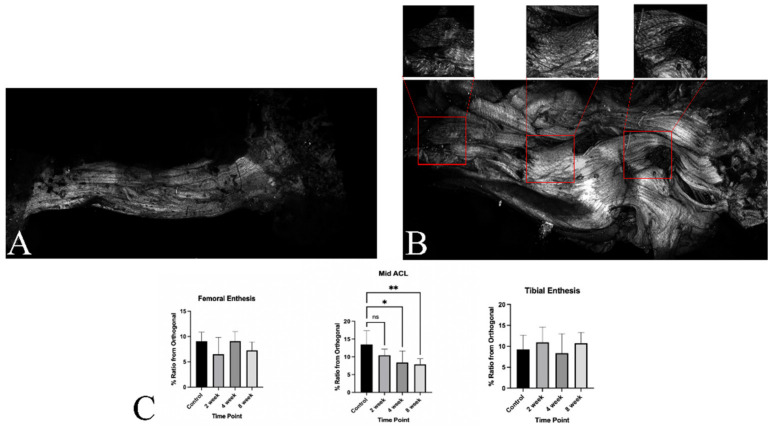
(**A**,**B**) Representative photos of multi-photon microscopy (MPM) of normal control anterior cruciate ligament (ACL) (**A**) and an 8 week specimen (**B**). 5× Whole ACL with punch out photos at 10X magnification. The regions in red boxes are showed in the photos above with magnification of 10×. (**C**) MPM quantitative results. *: *p* < 0.05, **: *p* < 0.01, ns: no significance.

**Figure 10:**
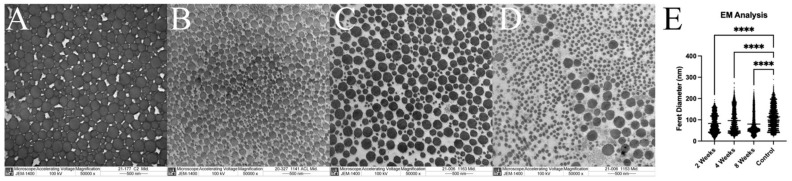
(**A**–**D**) Representative photos of transmission electron microscopy (TEM) of normal control anterior cruciate ligament (ACL) (Figure 10A) and surgical specimens at 2, 4, and 8 weeks (Figure 10B, Figure 10C and Figure 10D, respectively). (**E**) TEM quantitative results. ****: *p* < 0.0001.

Multi-photon microscopy demonstrated maintenance of the collagen fibril organization in the grafts. The native control ACL demonstrated a mean % deviation from the orthogonal value of 9.03 ± 1.81, 13.5 ± 3.83 and 9.24 ± 3.42 for the femoral enthesis, mid-ACL, and tibial enthesis, respectively. In comparison, the 2-week specimens had mean values of 6.50 ± 3.34 (femoral enthesis), 10.4 ± 1.73 (mid-ACL) and 10.97 ± 3.666 (tibial enthesis), with none being significantly different from the control. The 4-week samples had a mean of 9.10 ± 1.87 for femoral enthesis, 8.39 ± 3.28 for mid-ACL and 8.36 ± 4.65 for tibial enthesis. Lastly, the 8-week specimens demonstrated a mean deviation for the femoral enthesis of 7.26 ± 1.67, for the mid-ACL of 7.89 ± 1.58, and for the tibial enthesis of 10.8 ± 2.55. The values for the mid-ACL were significantly less than the controls at 4 and 8 weeks (*p* = 0.0126 and 0.0063, respectively), suggesting the maintenance of collagen fibril organization in the grafts. (Figure 9). Electron microscopy demonstrated significantly lower average collagen fibril diameter at all post-operative time points (82.83 ± 43.13 at 2 weeks group, 96.16 ± 55.14 at 4 weeks group and 79.99 ± 49.64 at 8 weeks group, respectively) compared to age-matched control ACLs (113.75 ± 53.65 at control group, *p* < 0.001) (Figure 10).

## 4. Discussion

The principal finding of this study is that B-ACL-B allograft reconstruction is feasible and can result in a normal anatomical appearance, with the maintenance of graft length and overall morphology. Furthermore, we found the progressive incorporation of the bone block into the host bone by new bone formation at the bone block–tunnel interface and the preservation of the native insertion site morphology with concomitant improvement in the biomechanical properties of the B-ACL-B allograft construct by 8 weeks. While some prior studies have evaluated bone plug healing in B-ACL-B allografts, the novelty of our study is the quantitative analysis of collagen fiber microstructure of the ligament matrix. Ultrastructural analysis demonstrated the maintenance of overall collagen matrix alignment, while there was repopulation with smaller diameter collagen fibrils, as would be expected during the process of graft incorporation and remodeling. The progressive healing suggests some degree of restoration of normal knee kinematics and supports the feasibility of replacement of the ACL with a bone–ACL–bone graft rather than the current standard of tendon graft reconstruction. 

The concept of “anatomic” ACL reconstruction has been popularized and has simply been related to graft placement at the anatomic tibial and femoral attachment sites of the native ACL. However, this is an over-simplification, as the standard tendon graft used for ACL reconstruction does not reproduce the complex microscopic anatomy of the native ACL, which is a highly complex tissue with a continuum of fibril lengths that reciprocally tighten and loosen throughout the range of knee motion, which contrasts with the largely single fiber bundle length of a tendon graft [18,19]. The rationale for the use of a B-ACL-B allograft is to restore the collagen fiber macro- and microstructure of the ACL. 

Histologic analysis of the B-ACL-B allograft demonstrated cell infiltration into the transplanted graft, consistent with the expected biologic process of graft incorporation. The infiltrating cells remodel the native ligament collagenous matrix and a produce newly synthesized matrix, potentially leading to the loss of macroscopic collagen fibril organization as demonstrated by the decreased birefringence at 8 weeks in the peripheral aspect of the graft in the areas of cellular infiltration. However, the multi-photon microscopy data suggests that there is generally the maintenance of overall collagen fiber organization. Furthermore, although matrix remodeling may diminish the material properties of the graft, the lack of difference in failure force and stiffness between the 4-week and 8-week specimens further supports the maintenance of overall structural properties of the grafts. The basic biological process of connective tissue allograft incorporation suggests that the collagen matrix of the allograft will be replaced by newly synthesized, small diameter collagen fibrils with inferior material properties compared to native ligament matrix. However, it is possible that the overall collagen fiber macrostructure of the graft will serve as a “template” to guide the alignment and organization of this new collagen matrix. Further studies in larger animal models will be important to examine the macrostructure of the collagen fiber matrix in the remodeled ligament.

In addition to the consideration of the ligament mid-substance, another important factor is that the complex structure and composition of the native ligament–bone enthesis is not re-established when a tendon graft is transplanted into a bone tunnel, as healing proceeds via the formation of a fibrovascular “scar tissue” interface that has inferior material properties [4,5,6,7]. Even when a bone–patellar tendon graft is used, there is typically tendon rather than bone at the tibial tunnel intra-articular aperture since the length of the patellar tendon (average 40–45 mm) is longer than the intra-articular length of the ACL, such that tendon–bone healing is required at the important tunnel aperture [3,20,21]. A B-ACL-B allograft will achieve fixation via direct bone-to-bone healing in both femoral and tibial tunnels. Our data demonstrate the maintenance of the microstructure of the native enthesis. 

The fixation of the B-ACL-B graft depends on bone-to-bone healing between the allograft bone block and the bone tunnel. μCT analysis demonstrated a gradual increase in the bone volume fraction at the bone block–bone tunnel interface over time, with healing proceeding mainly by direct (intramembranous) ossification. These findings are consistent with the normal biologic process of bone remodeling and incorporation, with osteoclastic bone resorption followed by osteoblastic new-bone formation [22]. Our finding of bone block pull-out as the location of failure during biomechanical testing suggests that graft incorporation in B-ACL-B allograft reconstruction was still incomplete at 8 weeks. Bone-to-bone healing in this model may be delayed due to both immunological response to allograft bone as well as a less rigid attachment due to suspensory fixation. Further evaluation in a larger animal model using direct aperture fixation with interference screws would add further insight into this ACL reconstruction technique. To reduce disease transmission and antigenicity, there are a variety of techniques to treat allografts, including deep freezing, sterilization by gamma irradiation, ethylene oxide, and proprietary chemical treatments. We chose deep-freezing at −80 °C due to prior findings that showed that the freezing process and duration of storage had no effect on the biomechanical or structural properties of ligaments [9,11,12,14]. However, the optimal technique to balance the reduction in antigenicity while maintaining graft mechanical properties needs to be determined in a future work. 

There were some limitations in this study. First, our observation was only 8 weeks, and therefore, we do not know the fate of the graft after continued remodeling is complete. A longer-term observation period is required to further understand the process of biologic incorporation. Second, since the biologic process of graft incorporation and remodeling is likely affected by the immunologic response to allograft tissue, more detailed characterization of the phenotype of the responding cells would provide further insight into the biologic process of graft healing. Another limitation is the uncontrolled mechanical loads on the healing graft post-operatively due to the inability to control weight bearing and knee motion. Thirdly, the suspensory sutures placed at the bone-ACL interface may weaken the graft biomechanical strength. However, considering the suture was very thin (2-0 Ethibond suture) and it was placed in each group, it may not affect the result of comparison between groups. Finally, because the rabbit ACL is too small to be measured accurately with calipers, we matched donor and recipient rabbits according to similar weights, which may increase the risk of graft size mismatch. However, we confirmed the isometric tension of the grafts with the full range of joint motion during surgery, as well as the proper placement of the graft using radiographic imaging and by macroscopic inspection post-surgery for each rabbit. Despite these limitations, we believe that this study provides a significant basis for understanding the behavior of B-ACL-B allografts in ACL reconstruction, and the rabbit model may be a promising tool for further research.

## 5. Conclusions

Our rabbit model of B-ACL-B allograft ACL reconstruction demonstrates the restoration of the normal macroscopic anatomy of the ACL, with progressive graft incorporation and remodeling. The B-ACL-B allograft may provide the closest possible structural and biomechanical match to the native ACL, resulting in improved functional outcomes and may serve as a future option for ACL reconstruction. 

## Data Availability

The datasets generated and analyzed during the current study are available from the corresponding author on reasonable request.
